# Freeze-thaw lysates of *Plasmodium falciparum*-infected red blood cells induce differentiation of functionally competent regulatory T cells from memory T cells

**DOI:** 10.1002/eji.201142164

**Published:** 2012-05-14

**Authors:** Olivia C Finney, Emma Lawrence, Alice P Gray, Madi Njie, Eleanor M Riley, Michael Walther

**Affiliations:** 1 Department of Immunology and Infection, Faculty of Infectious and Tropical Diseases, London School of Hygiene and Tropical MedicineKeppel Street, London, UK; 2 Malaria Programme, MRC LaboratoriesFajara, Banjul, The Gambia; 3 Faculty of Life Sciences, The University of Manchester, Simon BuildingBrunswick Street, Manchester, UK; 4 National Institute of Allergy and Infectious Diseases, NIHRockville, MD, USA

**Keywords:** Malaria Immunology, Memory T cells, Treg cells, Treg-cell induction

## Abstract

In addition to naturally occurring regulatory T (nTreg) cells derived from the thymus, functionally competent Treg cells can be induced in vitro from peripheral blood lymphocytes in response to TCR stimulation with cytokine costimulation. Using these artificial stimulation conditions, both naïve as well as memory CD4^+^ T cells can be converted into induced Treg (iTreg) cells, but the cellular origin of such iTreg cells in vivo or in response to more physiologic stimulation with pathogen-derived antigens is less clear. Here, we demonstrate that a freeze/thaw lysate of *Plasmodium falciparum* schizont extract (PfSE) can induce functionally competent Treg cells from peripheral lymphocytes in a time- and dose-dependent manner without the addition of exogenous costimulatory factors. The PfSE-mediated induction of Treg cells required the presence of nTreg cells in the starting culture. Further experiments mixing either memory or naïve T cells with antigen presenting cells and CFSE-labeled Treg cells identified CD4^+^CD45RO^+^CD25^−^ memory T cells rather than Treg cells as the primary source of PfSE-induced Treg cells. Taken together, these data suggest that in the presence of nTreg cells, PfSE induces memory T cells to convert into iTreg cells that subsequently expand alongside PfSE-induced effector T cells.

## Introduction

Two distinct populations of regulatory T (Treg) cells have been described: “natural” regulatory T (nTreg) cells that arise in the thymus and circulate in the blood 1, and “adaptive” or “induced” regulatory T (iTreg) cells, arising in the periphery in response to a specific antigen stimulus 2. The expansion of the Treg-cell population in the periphery during infection has been attributed to both proliferation of nTreg cells and conversion of CD4^+^CD25^−^ cells to iTreg cells 3b^4^. iTreg cells are thought to develop in peripheral lymphoid tissue in response to specific antigenic stimulation through the T-cell receptor (TCR), costimulatory receptors, and cytokine stimulation 5. For instance, anti-CD3/CD28 stimulation or exposure to antigen via immature DCs 6b^7^b^8^ has led to conversion of conventional T cells into iTreg cells, and culturing peripheral murine CD4^+^CD25^−^ T cells with a well-defined nonself antigen in the presence of TGF-β induces functional FOXP3^+^CD25^+^, antigen-specific Treg cells 4. Although this observation has been confirmed for human cells 9b^10^, the induction of iTreg cells by TGF-β in human cells remains controversial and depends on the antigen dose and the route of administration 11b^12^b^13^b^14^, suggesting that different mechanisms of induction may be relevant in different situations. In addition to TGF-β, an important role for IL-2 has been demonstrated for the induction of human iTreg cells 15. Furthermore, Zheng and colleagues 9 showed that the presence of thymus-derived nTreg cells can greatly enhance the cytokine-mediated induction of iTreg cells from human naïve peripheral CD4^+^CD25^−^ cells (Tnaive), and in mice, activated nTreg cells were shown to confer infectious tolerance in a TGF-β-dependent manner 16.

However, to date, in vitro studies on the induction of Treg cells from CD4^+^CD25^−^ T cells tended to employ nonphysiological stimuli such as TCR ligation via anti-CD3 in the presence of exogenously added recombinant cytokines. Using these conditions, iTreg cells can be induced from both naïve and memory T (Tmem) cells 17, but it is not at all clear which of these two T-cell populations is most relevant under physiologic conditions when antigen is encountered in vivo. There are, however, intriguing data derived ex vivo using deuterium-labeled glucose that suggest that Treg cells are renewed from a pool of Tmem cells 18. To further investigate the pathways of iTreg-cell induction that are most relevant to the in vivo situation will require studies using genuine antigens rather than artificial TCR agonists and costimulatory molecules.

First studied for their role in autoimmunity and tumor immunology 1b^19^b^20^, it is now clear that CD4^+^CD25^+^FOXP3^+^CD127^lo/−^ Treg cells also play a role in infectious diseases 3b^21^. They are, for instance, induced upon infection with malaria in several different murine models (reviewed in 22b^23^), and data from experimentally infected humans indicate that *Plasmodium falciparum* infection induces Treg cells in vivo and that this correlates with a transient increase in TGF-β 24. More recently, Scholzen et al. [30] have shown that intact, *P. falciparum*-infected RBCs induce Treg cells from proliferating CD4^+^ CD25^−^ cells in an IL-2, IL-10, and TGF-β dependent manner, which does not require contact with antigen presenting cells (APCs). However, the source of the CD4^+^ CD25^−^ cells (i.e. naïve or memory) was not determined.

Here, we present data confirming that Treg cells are activated and induced in vitro in response to malaria antigen in a time- and dose-dependent manner. These iTreg cells were functional in suppressing proliferation of CD4^+^CD25^−^ cells. Furthermore, iTreg cells were derived from Tmem cells and this process required the presence of nTreg cells, leading us to hypothesize that nTreg cells are able to convert Tmem cells into iTreg cells that subsequently expand in parallel to effector T (Teff) cells.

## Results

### Treg cells are induced in vitro in response to PfSE

**Figure 1:**
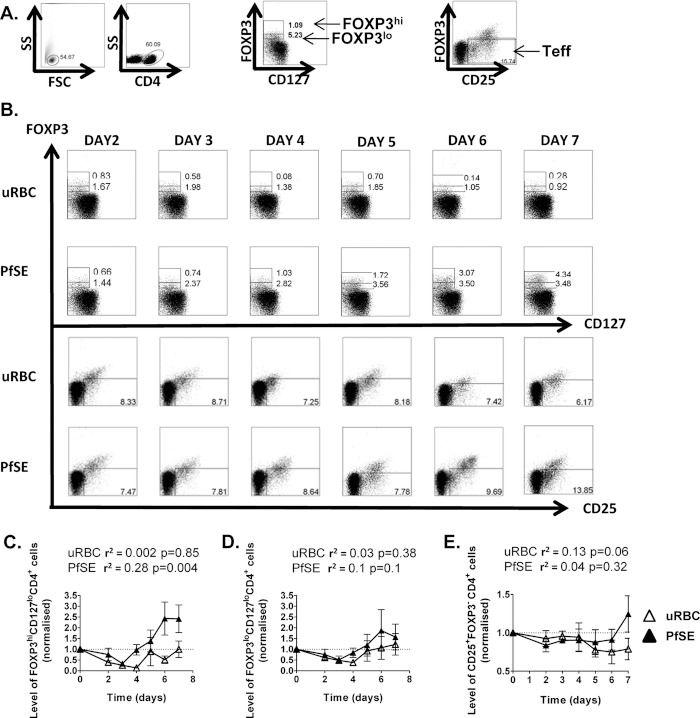
Treg cells are induced and activated by *P. falciparum* schizont extract (PfSE). PBMCs were cultured for up to 7 days with growth medium (GM), uninfected red blood cells (uRBCs), or PfSE (PfSE, at a 2 parasite:1 PBMC ratio, equivalent to 2 × 10^6^ parasites/mL). Cells were stained for CD4, CD25, CD127, and FOXP3 and the percentage of FOXP3^+^CD127^lo/−^ and CD25^+^FOXP3^−^CD4^+^ cells was determined ex vivo, and after 2, 3, 4, 5, 6, and 7 days of culture. (A) Lymphocytes were gated, followed by CD4^+^ cells. Treg cells were defined as CD4^+^FOXP3^+^CD127^lo/−^ cells. In order to draw this gate, cells were first gated on CD4^+^FOXP3^+^ cells, and the CD127^lo/+^ cutoff was determined on the CD4^+^FOXP3^+^ population, where two populations are clearly visible. These gates were then applied to CD4^+^ T cells, displayed as FOXP3 versus CD127. On this plot, FOXP3^+^CD127^lo/−^ cells were further divided into FOXP3^hi^ and FOXP3^lo^-expressing cells. T effector (Teff) cells were defined as CD4^+^CD25^+^FOXP3^−^. (B) Representative stainings of PBMCs from one of five donors, treated and gated as in (A). The kinetics of (C) CD4^+^FOXP3^hi^CD127^lo/−^, (D) CD4^+^FOXP3^lo^CD127^lo/−^Treg, and (E) Teff cells (CD4^+^CD25^+^FOXP3^−^) in response to uRBCs and PfSE are shown. Expression levels on day 0 were set to “1”, and values measured on subsequent days were expressed as a fold change compared to day 0. Data are based on the collection of 50,000 lymphocytes. Data are shown as mean ± SEM of values from five donors (each tested twice in independent experiments). *R* values and *p* values are derived from regression analysis.

To investigate whether *P. falciparum* antigens can induce Treg cells in vitro, peripheral blood mononuclear cells (PBMCs) from five healthy malaria-naïve donors, each assayed twice, were cultured for 7 days with growth medium (GM), uninfected red blood cells (uRBCs), or *P. falciparum* schizont extract (PfSE) and the percentage of CD4^+^ cells expressing a Treg-cell (FOXP3^+^CD127^lo/−^) or Teff-cell phenotype (defined as CD4^+^CD25^+^FOXP3^−^ cells) was determined ex vivo, and on days 2, 3, 4, 5, 6, and 7 by flow cyto-metry (Fig. 1A and B). Considering that suppressive capacity is predominantly found in FOXP3^hi^-expressing CD4^+^CD127^lo/−^ cells 25, CD4^+^CD127^lo/−^ cells were further subdivided into FOXP3^hi^ and FOXP3^lo^-expressing cells. Values were normalized setting the percentage of cells determined ex vivo as the baseline value of 1.

The percentage of Treg cells (both FOXP3^hi^ and FOXP3^lo^) among CD4^+^CD127^lo/−^ T cells that were unstimulated (GM; data not shown) or cultured with uRBCs (Fig. 1C and D) was stable throughout the 7 days of the experiment. In contrast, among cells cultured with PfSE, the percentage of FOXP3^hi^ Treg cells increased steadily from day 4 (Fig. 1B and C) and was, on average, 2.4-fold higher on day 6 than on day 0. The percentage of FOXP3^lo^CD4^+^CD127^lo/−^ T cells showed a slight, albeit nonsignificant increase in response to PfSE stimulation. While we cannot formally rule out the possibility that the increasing proportion of Treg cells over time in PfSE-stimulated cultures is simply due to death of non-Treg cells, we consider this to be unlikely, since the proportion of Teff cells among CD4^+^ T cells did not drop significantly, but showed a tendency to increase toward the end of the culture period (Fig. 1E).

### Treg cells are induced in a dose-dependent fashion

**Figure 2:**
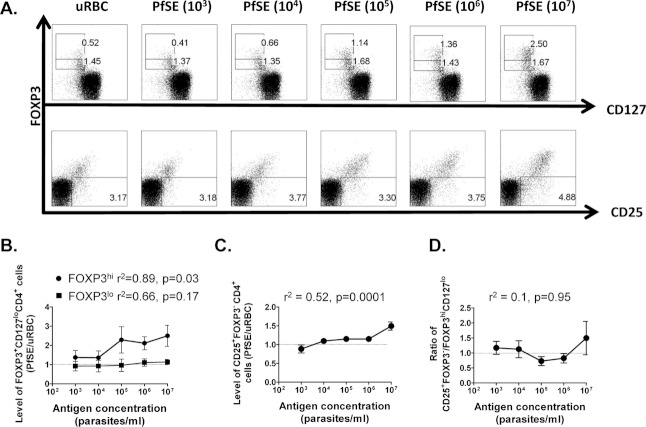
Treg cells are induced and activated by PfSE in a dose-dependent manner. PBMCs were cultured for 6 days with different concentrations of PfSE (10^3^, 10^4^, 10^5^, 10^6^, 10^7^ parasites/mL). Uninfected red blood cells were used as a control. On day 6, cells were stained for CD4, CD25, CD127, and FOXP3. (A) Representative staining using the gating strategy defined in Fig. 1A. The level of (B) FOXP3^hi^ (circles) and FOXP3^lo^ (squares) CD4^+^FOXP3^+^CD127^lo/−^ cells, and (C) T effector cells (CD4^+^CD25^+^FOXP3^−^) is shown, expressed as the ratio of cells measured in PfSE/uRBC stimulated wells. In (D), the ratio of Teff:CD4^+^FOXP3^hi^CD127^lo/−^ cells is shown. Data are based on the collection of 50,000 lymphocytes. Data are shown as ± SEM from five donors, each tested twice in independent experiments. *R* values and *p* values derived from regression analysis.

We hypothesized that, if the induction of Treg cells is driven by *P. falciparum* antigen, Treg-cell frequencies might increase with increasing antigen dose. Cells were cultured for 6 days with five different concentrations of PfSE (10^3^–10^7^ parasites/mL), equivalent to parasitaemia levels commonly encountered in acute disease, and the percentage of FOXP3^hi^ and FOXP3^lo^ CD4^+^CD127^lo/–^ cells and Teff cells was determined. GM and uRBCs (at equivalent doses to PfSE) were used as controls (Fig. 2A). Data are expressed as the ratio of cells in PfSE cultures/uRBC cultures. The percentage of both, FOXP3^hi^CD4^+^CD127^lo/–^ cells (Fig. 2B) as well as the percentage of Teff cells (Fig. 2C) increased significantly with increasing antigen concentration, resulting in a stable ratio of Teff:FOXP3^hi^CD4^+^CD127^lo/–^ cells (Fig. 2D), while the percentage of FOXP3^lo^CD4^+^CD127^lo/–^ cells did not vary significantly. Taken together, and in line with our observations made ex vivo 26, this suggests that PfSE-induced upregulation of Teff is balanced by a commensurate increase in iTreg cells, expressing high levels of FOXP3. However, the ratio of Teff to FOXP3^hi^ Treg cells did increase at the highest antigen concentration (10^7^ parasites/mL), suggesting that — as we have observed in children with acute clinical malaria 27 — at higher concentrations of antigen, the coregulation of Teff and Treg cells may fail.

### PfSE-induced Treg cells are functionally suppressive

Since it has been shown that activated Teff can transiently upregulate FOXP3 in culture without acquiring suppressive function 28, it is important to verify whether the PfSE-induced cells expressing a Treg cell phenotype were indeed functional Treg cells. Following an approach described by Hisaeda 29, cells were cultured for 6 days in GM, uRBCs, or PfSE. On day 6, CD25^+^ and CD25^−^ cells were purified using CD25 microbeads, recombined at varying CD25^+^:CD25^−^ ratios of 0:1, 1:20, 1:4, 1:1, cultured for a further 3 days with anti-CD3/anti-CD28 antibodies, and their proliferation analyzed by ^3^H-thymidine incorporation since only approximately 25% (median 27.2%, IQR 11.1 – 37.5) of CD25^+^ selected cells actually express the CD127^lo/−^ FOXP3^+^ phenotype, the true ratio of Treg cells:non-Treg cells in these cultures was in fact much lower (approximately 0:1, 1:76, 1:19, 1:7)).

**Figure 3:**
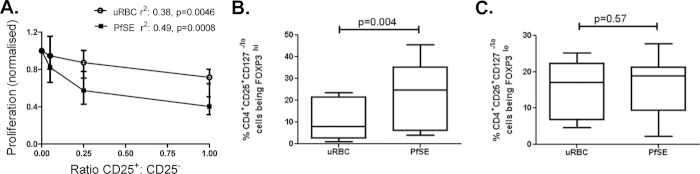
Induced Treg cells suppress proliferation. PBMCs were cultured for 6 days with GM, uRBCs, or PfSE. Cells were separated into CD25^+^ and CD25^−^ PBMCs using Miltenyi CD25 beads. CD25^+^ and CD25^−^ were cultured in triplicate wells in 96 well plates in different ratios (0:1, 1:20, 1:4, 1:1) and incubated with CD3/CD28. Cells were incubated for a further 18 h with ^3^H-thymidine before being analyzed in a scintillation counter. The proliferation was measured as counts per minute, and normalized on proliferation in wells containing CD25^−^ cells only. (A) The median and interquartile range of values from five donors (each tested twice in independent experiments) is shown. *R* values and *p* values derived from linear regression analysis (df = 18). The proportion of CD4^+^CD25^+^CD127^lo/−^ cells among isolated CD25^+^ cells expressing either (B) high or (C) low levels of FOXP3 is shown for uRBC- and PfSE-stimulated cultures. Box plots show median and interquartile ranges with whiskers indicating the 5 and 95% percentiles from five donors, each tested twice in independent experiments.

Despite the modest purity of Treg cells achieved with this isolation method, increasing the ratio of CD25^+^ to CD25^−^ cells significantly reduced the proliferative response to anti-CD3/anti-CD28 in the GM (data not shown) or uRBC-stimulated cultures (Fig. 3A). In the PfSE-stimulated cultures, this effect was even more pronounced. The CD25^+^ cells isolated from PfSE-stimulated cultures contained significantly more FOXP3^hi^ cells than CD25^+^ cells isolated from uRBC-stimulated cultures (*p* = 0.004, Fig. 3B), while the percentage of FOXP3^lo^ cells was similar in CD25^+^ cells isolated from both culture conditions (*p* = 0.57, Fig. 3C). Taken together, this suggests that the preferential induction of FOXP3^hi^ cells in response to PfSE stimulation is responsible for the enhanced suppression caused by CD25^+^ cells isolated from PfSE-stimulated cultures.

### Induction of Treg cells by PfSE requires Treg cells in the primary culture

It has been established previously that upregulation of Treg cells in response to stimulation with intact infected red blood cells (iRBCs) requires activated monocytes, as well as IL-2, IL-10, and TGF-β 30. In these experiments, Treg cells were predominantly derived from CD25^−^FOXP3^−^CD4^+^ T cells, raising the intriguing question whether memory or naïve T cells can convert into Treg cells as previously suggested 4b^18^, and whether the presence of Treg cells is required to initiate this process. To further explore the cellular requirements for PfSE-mediated Treg cell induction, CD25^+^ cells were depleted from freshly isolated PBMCs that were then cultured for 6 days with GM, uRBCs, or PfSE; mock-depleted cells were used as a control.

**Figure 4:**
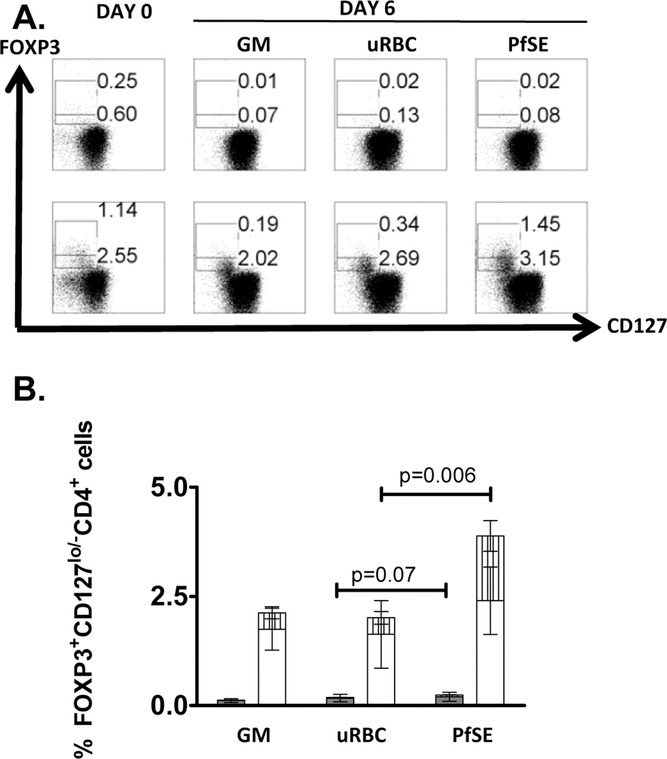
Depletion of nTreg cells abrogates the induction of Treg cells. PBMCs were depleted of CD25^+^ cells using Miltenyi CD25 beads (depletion of 96.41% [CI 95% 95.25–97.57], resulting in 75.78% (CI 95% 66.42–85.13) Treg-cell depleted). CD25^−^ PBMCs and mock-depleted PBMCs were cultured for 6 days with GM, uRBCs, and PfSE. On day 6, cells were stained with CD4, CD25, CD127, and FOXP3. (A) Representative staining of CD25^−^-depleted (top panel) and mock-depleted (bottom panel) cells. (B) After 6 days of culture, the percentage of CD4^+^FOXP3^+^CD127^lo/−^ cells was determined in the depleted (filled bars) and the mock-depleted cells (open bars). The white area of the bar represents the proportion of CD4^+^FOXP3^lo^CD127^lo/−^cells, the striped area represents the proportion of CD4^+^FOXP3^hi^CD127^lo/-^cells. Data are based on the collection of 50,000 lymphocytes. Data are shown as ± SEM from five donors, each tested twice in independent experiments. *p* values calculated using the Mann–Whitney test.

In the mock-depleted samples, as expected, the proportion of Treg cells among CD4^+^ cells increased significantly from day 0 to day 6 in the PfSE-stimulated cultures and was significantly higher than in the control cultures (Fig. 4). As demonstrated in Fig. 1, this increase was predominantly driven by a 3.9-fold increase of FOXP3^hi^CD4^+^CD127^lo/−^ cells, compared to a 1.4-fold increase in FOXP3^lo^-expressing cells. However, in the CD25-depleted PBMC cultures, the proportion of Treg cells remained low throughout the culture period and there was no significant change in the proportion of Treg cells over time or between culture conditions. We conclude, therefore, that the PfSE-mediated increase in Treg cells is either due to proliferation of preexisting CD25^+^ nTreg cells, or that conversion of CD25^−^ cells into iTreg cells requires the presence of CD25^+^ nTreg cells in the culture.

### Memory T cells convert into Treg cells upon stimulation with PfSE

To further investigate whether the increase in Treg cells seen in response to PfSE stimulation was primarily due to proliferation of preexisting Treg cells or to conversion of naïve and/or Tmem cells into FOXP3-expressing cells, we designed an experiment where either Tmem or Tnaïve cells depleted of Treg cells were mixed with CFSE-labeled Treg cells and stimulated for 5 days with uRBCs or PfSE in the presence of APCs (see Supporting Information Fig. 1 for experimental design, and purities of cell subsets). Blood was obtained from three adult donors who had a confirmed episode of *P. falciparum* infection in the last 5 months.

**Figure 5:**
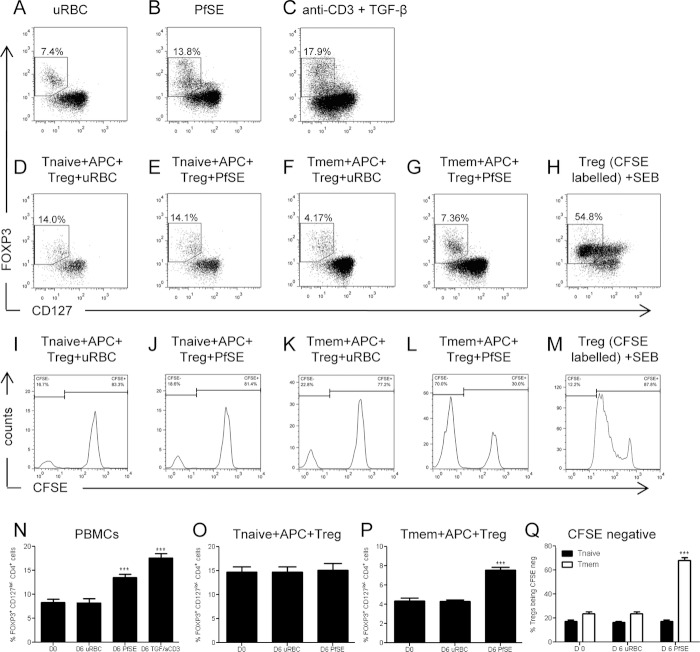
PfSE stimulation induces iTreg cells from Tmem cells. The proportion of viable CD4^+^ T cells expressing a Treg-cell phenotype after 5 days culture is shown for PBMCs stimulated with (A) uRBCs, (B) PfSE, (C) anti-CD3, and TGFβ, for the cell mix containing “Tnaive” + “APC” + “Treg”, stimulated with (D) uRBCs, (E) PfSE; for the cell mix containing “Tmem” + “APC” + “Treg,” stimulated with (F) uRBCs, (G)PfSE; and for (H) CFSE-labeled “Treg” stimulated with SEB. (I–M) The histograms depict the proportion of CFSE+/− cells expressing a Treg-cell phenotype gated in the corresponding graphs (D–H). A summary of the proportion of viable CD4^+^ T cells expressing a Treg-cell phenotype prior to and after culture is shown for (N) PBMCs, (O) the cell mix containing “Tnaive” + “APC” + “Treg cells” and (P) “Tmem” + “APC” + “Treg cells.” (Q) The proportion of CFSE-negative cells expressing a Treg-cell phenotype prior to and after culture is shown for the cell mixes containing either naïve or Tmem cells. Data are shown as mean ± SEM. Representative plots from one of three experiments are shown in A to H.

PBMCs were incubated for 5 days with uRBCs (Fig. 5A), or PfSE (Fig. 5B). In addition, PBMCs were stimulated with anti-CD3 and TGF-β (Fig. 5C), and CFSE-labeled enriched “Treg” were stimulated with staphylococcus enterotoxin B (SEB) (Fig. 5H). Representative examples for the proportion of CD4^+^ T cells expressing a Treg-cell phenotype in the cell mix containing “Tnaive + APC + Treg” are depicted in Fig. 5D (uRBC stimulated) and E (PfSE stimulated), and for the cell mix containing “Tmem + APC + Treg”, examples are shown in Fig. 5F (uRBC stimulated) and G (PfSE stimulated). Stimulation with either PfSE or TGF-β/anti-CD3 induced significant expansion of Treg cells in PBMCs (Fig. 5N). While the proportion of CD4^+^ T cells expressing a Treg-cell phenotype in the “Tnaive mix” cultures did not change during the culture period (Fig. 5O), the proportion of Treg cells in the “Tmem mix” almost doubled in response to stimulation with PfSE, being significantly higher as compared to both Treg cells in the “Tmem mix” on day 0 or after stimulation with uRBCs (Fig. 5P). This indicates that PfSE-mediated induction of Treg cells requires the presence of Tmem but not Tnaïve cells. The histograms showing the proportion of Treg-cell staining for CFSE in the various cell mixes (defined in Fig. 5D–H) after the culture period are shown in Fig. 5I–M, and reveal that proliferation of cells with a Treg-cell phenotype (as indicated by sequential dilution of CFSE) was only observed for SEB-stimulated preexisting “Treg” (Fig. 5M). However, the proportion of cells expressing a Treg-cell phenotype that are CFSE negative increased significantly in the PfSE-stimulated “Tmem mix” (Fig. 5L) from a mean of 23.6% on day 0 to a mean of 68.3% on day 5 (Fig. 5Q). While it could be argued that stimulation with PfSE resulted in extensive proliferation of Treg cells (i.e. completely diluting out the CFSE), we consider this to be highly unlikely—given that even stimulation of “Treg” with SEB (Fig. 5M) did not result in complete dilution of CFSE. Taken together, therefore, these data are compatible with the hypothesis that PfSE-mediated induction of Treg cells is primarily due to a conversion of Tmem cells into iTreg cells.

## Discussion

The induction of Treg cells in response to TCR stimulation has been studied in a variety of experimental models and diseases both in mice and men, and a variety of mediators essential to this process has been identified. However, the cellular origin of Treg cells expanding in response to an antigenic stimulus is not entirely clear. An obvious possibility is that Treg cells might regenerate continuously from pre-existing Treg cells throughout life. Indeed, Treg cells have now been shown to proliferate rapidly in humans in vivo 31. However, the observation that Treg cells are — in contrast to Tmem cells — highly proapoptotic 26b^32^b^33^b^34^, would imply that Treg cells must have an extraordinarily high regenerative potential if this was their primary mechanism of maintenance over the duration of a human life span. But, in fact, their capacity for repeated self-regeneration is limited 18: In contrast to Tmem cells, Treg cells lack the ability to upregulate telomerase activity, which makes them prone to telomere erosion 31b^35^. An additional mechanism for Treg-cell expansion during an immune response — other than exclusive proliferation of preexisting Treg cells — is thus likely. Several studies have demonstrated that Treg cells can be induced alongside Teff cells from human CD4^+^ CD25^−^ T cells in response to stimulation with CD3/CD28 and the appropriate cytokine cocktail 11, with some studies identifying the source as either Tnaive cells 9, or both Tnaive and Tmem cells 17. Stimulation of CD4^+^CD25^−^ T cells with intact *P. falciparum*-infected RBCs led to induction of Treg cells 30, but this study did not define the phenotype of the CD4^+^CD25^−^ T cells giving rise to the Treg cells.

The observation that Treg and Tmem cells display, on average, 80% homology in their TCR Vβ usage 35b^36^b^37^ implies close relatedness between these two cells and has prompted the hypothesis that in vivo, a substantial proportion of Treg cells induced in response to antigenic stimulation might be derived from conversion of Tmem into Treg cells 18b^31^. The data presented in here are among the first to demonstrate that Tmem cells can indeed give rise to cells expressing a Treg-cell phenotype (CD4^+^CD127^lo/−^FOXP3^+^) in response to stimulation with a clinically relevant antigen without exogenously added costimulatory factors, and further indicate that proliferation of preexisting Treg cells makes little if any contribution to the antigen-induced increase in Treg cells.

Nevertheless, we noticed that the PfSE-mediated induction of Treg cells was dependent on the presence of Treg cells in the starting culture. This is in agreement with the results of Scholzen et al., who observed that intact *P. falciparum*-infected RBCs induced substantially fewer Treg cells from CD25-depleted PBMCs than from whole PBMCs 30. Considering that these experiments were carried out using blood from malaria-naïve individuals, it can be assumed the Treg cells required to induce this process are naturally occurring nTreg cells. In our mixing experiments, CFSE-labeled Treg cells did not proliferate in response to PfSE, confirming that proliferation of preexisting Treg cells is not the primary explanation of the increase in Treg cells in PfSE cultures and suggesting they may serve another function. Our data are reminiscent of “infectious tolerance” 38 where nTreg cells were shown to confer suppressive activity onto conventional CD4^+^ T cells that were subsequently shown to develop a Treg cell phenotype including FOXP3 expression 39. More recent studies have begun to unravel the molecular mechanism of infectious tolerance, and how the malaria parasite might regulate this process. In mice, TGF-β bound to its latency-associated peptide is present on activated nTreg cells. Once converted into its bioactive from, membrane-bound TGF-β was shown to exert a paracrine effect on neighboring T cells, inducing them to become iTreg cells 16. A recent elegant study demonstrated that PfSE converted latent TGF-β bound to the membrane of activated human Treg cells into its bioactive form, resulting in the expansion of Treg cells 40. Taken together, it is tempting to speculate that malaria parasites might initiate the induction of iTreg cells by activating membrane-bound TGF-β on nTreg cells that exerts a paracrine effect on Tmem cells, converting them into iTreg cells. Another avenue worth exploring when studying the mechanism of infectious tolerance is the role of IL-35, considering that Treg cells were shown recently to confer suppressive activity to conventional T cells in an IL-35 dependent manner 41.

Although several studies report a transient upregulation of FOXP3 in activated human Teff CD4^+^ cells that is not associated with the acquisition of suppressive function 28b^42^b^43^, it is highly unlikely that Teff activation explains our results. Firstly, we established that the FOXP3^+^ cells induced by PfSE stimulation are functionally competent and able, in a dose-dependent manner, to suppress proliferation of Teff cells. Moreover, the transient FOXP3 expression described for human Teff cells peaked at day 3 and declined rapidly thereafter 42b^43^b^44^, while we performed our analysis on cells cultured for at least 5 days. Most importantly, the transient increase of FOXP3 in these studies was not associated with the acquisition of a Treg cell phenotype 44, and FOXP3 expressing Teff cells maintained CD127 expression 42, while we defined PfSE-induced Treg cells stringently as CD127^lo/−^-expressing cells.

**Figure 6:**
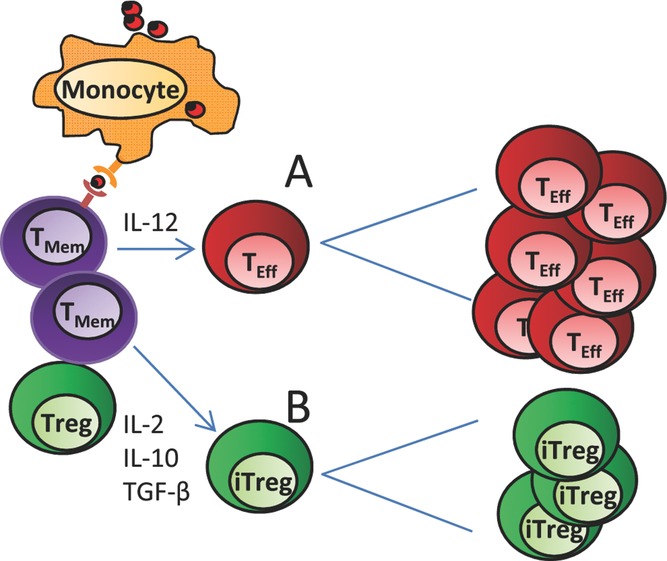
Model for induction of Treg cells in response to antigenic stimulation. In the appropriate cytokine environment, and upon presentation of their cognate antigen memory T (Tmem) cells convert into (A) effector T (Teff) cells, or (B) induced Treg (iTreg) cells. The formation of the latter requires contact of the Tmem cells with a preexisting Treg cells (which may involve membrane-bound TGF-β, activated by the parasite). Both Teff and iTreg cells subsequently proliferate in a balanced manner.

An intriguing question is how Tmem-derived Treg cells expand. Do large fractions of Tmem cells simply convert into iTreg cells, or do converted Treg cells subsequently expand by proliferation? When whole PBMCs were labeled with CFSE and stimulated with intact iRBCs, Scholzen et al. observed 30 an initial upregulation of FOXP3 expression for the first 3 days in the absence of proliferation that was then followed by proliferation of FOXP3^+^ cells. In a variation of that approach, we directly labeled purified Treg cells with PfSE prior to putting them into culture with unlabeled Tmem cells. While we find no evidence for proliferation of the Treg cells that were added initially, we do observe a population of non-CFSE-labeled Treg cells emerging from the Tmem-cell population. When interpreted together, these experiments suggest that upon encounter with the antigen, some Tmem cells are converted into iTreg cells and these iTreg cells subsequently expand by proliferation. As discussed above, the initiation of this process seems to require the presence of CD25^+^ cells that could be nTreg cells (see Fig. 6). While we and others 30 have demonstrated that Treg cells generated by stimulation of PBMCs with malarial antigens are suppressive, future experiments need to formally establish the suppressive capacity of Treg cells derived from Tmem cells in response to PfSE stimulation. In this respect, better strategies to obtain highly purified CD4^+^CD127^−/lo^FOXP3^+^ T cells would be most desirable. While the approach based on CD25 chosen in this study may be sufficient to deplete CD4^+^CD127^−/lo^FOXP3^+^ T cells, more sophisticated mechanistic studies on malaria-induced Treg cells will require highly purified Treg populations that cannot be achieved with the approach chosen here. Our data also suggest that Teff and Treg cells increase in parallel in response to PfSE stimulation in vitro. This is in line with the kinetics of Treg and Teff cells we have described previously, ex vivo, for individuals exposed to seasonal malaria 26, and consistent to a previous report where a parallel increase in Treg and Teff cells was observed for cells derived from a skin blister after intradermal injection of purified protein derivative of tuberculin (PPD) to BCG-vaccinated individuals 43. These examples of a tightly regulated expansion of Teff and Treg cells lend support to the hypothesis that in response to an antigenic stimulus, Tmem clones give rise to both Teff- and Treg-cell populations that subsequently expand 18, most probably in response to the presence of cytokines such as IL-2, in order to maintain immune homeostasis.

## Materials and methods

### Donors and preparation of cells

Donors were recruited from the expatriate work force at the Medical Research Council (MRC) Laboratories, The Gambia. All donors were healthy, and unless indicated otherwise, malaria naive. Blood was collected after written informed consent was obtained. Ethical approval was given by the Gambian Government/MRC Joint Ethics Committee and by the London School of Hygiene and Tropical Medicine Ethics Committee, United Kingdom.

For the studies on malaria-naïve individuals, up to 30 mL of venous blood was collected into heparinized vacutainers (BD). The blood was diluted in an equal volume of RPMI 1640, and PBMCs were isolated by density centrifugation using Nycoprep (Sigma-Aldrich). PBMCs were washed twice in RPMI 1640, and resuspended at 10^6^ cells/mL in GM (RPMI 1640, 10% AB^+^ serum (Sigma-Aldrich), 100 U/mL penicillin (Sigma-Aldrich), 100 μg/mL streptomycin (Sigma-Aldrich), 2 mM L-glutamine (Invitrogen Life Technologies)). Cells were then immediately stained for flow cytometry, or put into culture.

### Cell surface and intracellular staining for flow cytometry

Fresh or cultured cells were washed twice in FACS buffer (1× PBS, 2mM EDTA, and 1% FBS (Invitrogen Life Technologies)), and stained for surface markers at 4°C in the dark for 30 min. Intracellular staining was performed following the FOXP3 staining protocol (Ebioscience). The following Abs were used: CD4 PerCP, CD25 FITC, IgG1 PerCP, IgG1 FITC (all BD Biosystems), CD127 PE, FOXP3 allophycocyanin (clone PCH101), IgG1 PE, rat IgG2A allophycocyanin (all from Ebioscience). Samples were acquired on a CyanTM ADP flow cytometer using Summit software (Dako). Analysis was performed using FlowJo (Tree Star Inc.).

### Cell separations

CD25 depletion was achieved using CD25 microbeads according to the manufacturer's instructions (Miltenyi Biotech, Germany). Depletion removed on average 95% (95% CI: 93–97%) of CD25^+^ cells, resulting in a depletion of 76% (95% CI: 66–85%) of CD4^+^FOXP3^+^CD127^−/lo^ cells. Cells were mock depleted by adding cells to the matrix column without prior incubation with CD25 microbeads. CD25^+^ cells were recovered from the column according to the manufacturer's instructions.

CD4^+^CD45RA^+^ naïve T cells were isolated using the naïve T-cell isolation kit (Miltenyi Biotech) according to the manufacturer's instructions. A purity of 68% (95% CI: 56–90%) was obtained.

### Preparation of malarial antigens

*P. falciparum* parasites of the clone 3D7 were grown following the methods of Trager and Jensen 45 in O^+^ human erythrocytes from healthy donors in RPMI (Invitrogen, Life Technologies), supplemented with 25 mM HEPES (Sigma-Aldrich), 28 mM sodium bicarbonate (BDH), 20 ug/L hypoxanthine (Sigma-Aldrich), 5% albumax, and 5% human AB^+^ serum (Sigma). Cultures were gassed with 3% O_2_, 4% CO_2_, and 93% N_2_, and incubated at 37°C. The culture medium was changed daily and the parasitaemia was determined by examination of Giemsa-stained thin blood smears. Mature schizonts were harvested from cultures of 5–8% parasitaemia by centrifugation through a Percoll/Sorbitol gradient (Sigma-Aldrich, >95% purity). PfSE was prepared by two rapid freeze-thaw cycles in liquid nitrogen and a 37°C water bath. All cultures were routinely shown to by Mycoplasma free by PCR (Bio Whittaker).

### Cell culture

Cells were cultured in 48 well plates for a maximum of 7 days. PfSE was used at a concentration equivalent to two iRBCs per mononuclear cell (PBMC). A similar freeze thaw preparation of uRBCs was used as a control. GM was used as a negative control. For dose-dependence assays, PfSE was used in five different concentrations (10^3^, 10^4^, 10^5^, 10^6^, 10^7^ parasites per mL).

### Treg-cell suppression assay

Cells were cultured in 24 well plates for 6 days with PfSE. uRBCs and GM were used as negative controls. On day 6, CD25^+^ cells were depleted from PBMCs using anti-CD25 mAb-conjugated microbeads as described above. The phenotype of cell fractions was analyzed by flow cytometry as described above. CD25^+^ and CD25^−^ cells were cultured in triplicate wells in 96 well plates for 3 days with soluble anti-CD3 and anti-CD28 at ratios of 0:1, 1:20, 1:4, and 1:1 CD25^+^:CD25^−^ cells.

On day 3, 1 μCi of ^3^H-thymidine (Amersham, UK) was added to each well. Eighteen hours later, cells were harvested onto cellulose filters, and tritium incorporation was measured by liquid scintillation counting (MicroBeta Counter, PerkinElmer).

### T-cell conversion assay

In a separate series of experiments, PBMCs from three expatriate donors with a documented episode of malaria in the last 5 months were used to address whether the increase in Treg cells seen in response to PfSE stimulation was due to proliferation of preexisting Treg cells or the induction of a Treg-cell phenotype in Tmem cells (see Supporting Information Fig. 1A for experimental setup). Using magnetically labeled beads from Miltenyi biotech according to the manufacturer's instructions, PBMCs were depleted of CD45RA^+^ and CD25^+^ cells to obtain a population enriched for Tmem cells and depleted of naïve T cells and Treg cells. Naïve CD45RA^+^ T cells (Tnaive cells) were isolated from PBMCs, previously depleted of CD25^+^ cells. APCs were prepared by positive selection of HLADR^+^ cells from PBMCs that were subsequently depleted of CD4^+^ cells to avoid contamination with Treg cells and other CD4^+^ T cells. To enrich Treg cells, CD25^hi^ cells were positively selected from PBMCs. Treg cells were subsequently labeled with 1 μM CFSE (Cell Trace, Invitrogen) following a previously described protocol 46. The purity of cell fractions was determined by flow cytometry using the following surface marker antibodies: anti-CD127 (PE), anti-CD45RA (Pe-Cy7), anti-HLA-DR (allophycocyanin-EFluor, all Ebioscience), anti-CD45RO (ECD, from Beckman Coulter), anti-CD4 (PerCP), and anti-CD25 (allophycocyanin, both from BD Biosystems), and the LIVE/DEAD fixable aqua dead cell stain kit from Invitrogen. Intracellular staining for FOXP3 was performed according to the Ebioscience protocol using anti-FOXP3 (Efluor 450). Appropriate isotype controls were used, and samples were acquired and analyzed as described above. Subsequently, 960,000 Tmem or Tnaive cells were mixed with 100,000 APC and 40,000 CFSE-labeled Treg cells. The mix was analyzed by flow cytometry to determine the proportion of CFSE-labeled Treg cells, using the panels described above. Untouched PBMCs and the cell mixtures were incubated for 5 days at 37°C and 5% CO_2_ with PfSE or uRBCs at a concentration equivalent to a ratio of five RBCs per one PBMC. In addition, PBMCs were stimulated with anti-CD3 (10 μg/mL, from BD) and TGF-β (2 ng/mL, from R&D systems, as described 4), and CFSE-labeled enriched Treg cells were stimulated with SEB at 2 ug/mL. After 5 days, cells were harvested and stained for flow cytometric analysis, using the panel described above.

### Statistical analysis

Statistical analyses were performed using Prism 4 software or STATA 10 software. Normal distribution was assessed using the D'Agostino & Pearson omnibus normality test. Where data were assessed to be normally distributed, parametric tests such as paired *t*-test and regression analysis using the Peason's correlation test was performed; for nonnormally distributed data, the Mann–Whitney test was used. When more than two groups were compared, a one-way repeated ANOVA was performed, with Dunnett post test adjusting for multiple comparisons.

## Abbreviations

GM: growth medium
iRBC: infected red blood cell
PfSE: *Plasmodium falciparum* schizont extract
Tmem cell: memory T cell
Tnaive cell: naïve T cell
uRBC: uninfected red blood cell

## Supplementary material

Disclaimer: Supplementary materials have been peer-reviewed but not copyedited.

**Suppl. figure 1 Experimental design and cell purities** A scheme of the experimental approach is depicted in Figure (A). Viable CD4+ T cells amongst (B): PBMC, (D): ‘Tmem’, (F): ‘Tnaive’ are shown according to their expression of memory markers CD45RA/RO, and according to their expression of Treg markers FOXP3 and CD127 (C: PBMC, E: ‘Tmem’, G: ‘Tnaive’, I: ‘APC’ and K: ‘Treg’). Compared to undepleted PBMC ‘Tmem’ were depleted of > 98% of CD4+ CD45RA+ and > 74% of CD4+ FOXP3+ CD127lo/- T cells. ‘Tnaive’ cells were depleted > 97% of CD4+ CD45RO+ and > 80% of CD4+ FOXP3+ CD127lo/- T cells compared to PBMC. (H): the percentage of viable cells expressing HLA-DR amongst ‘APC’ is shown. The ‘APC’ fraction comprised of a more than 77% pure HLADR+ population, of which > 94% of CD4+ FOXP3+ CD127lo/- T cells were depleted compared to PBMC. (J): Isolated ‘Treg’ were uniformly labeled with CFSE and contained at least 54% CD4+ FOXP3+ CD127lo/- cells (K). Note, for better comparability, percentages depicted in figures B-K refer to the proportion amongst viable cells, not CD4+ cells. The proportion of viable CD4+ T cells expressing a Treg phenotype in the cell mix containing (L): ‘Tnaive’ + ‘APC’ + ‘Treg’, or (N): ‘Tmem’ + ‘APC’ + ‘Treg’ is shown; The CFSE+ cells are depicted in blue, CFSE- cells are shown in red. The histograms in (M) and (O) show the proportion of CFSE+/- cells amongst viable CD4+FOXP3+CD127lo/- cells shown in (L) and (N). ‘Tnaive’ mix (L) and ‘Tmem’ mix (N) contained a mean of 14.73% and 4.35% CD4 cells expressing a Treg phenotype, respectively, out of which a mean of 17.6% (in the mix with ‘Tnaive’) and 23.6% (in the mix with ‘Tmem’) were CFSE neg, indicating the presence of contaminating Treg from either of the non-labelled T cell compartments (‘Tmem’ or ‘Tnaive’) or added ‘APC’.
