# Supplementary material for: Freeze-thaw lysates of Plasmodium falciparum-infected red blood cells induce differentiation of functionally competent regulatory T cells from memory T cells
Source: Eur J Immunol. 2012 May 14;42(7):1767–77. doi: 10.1002/eji.201142164 (PMC3549566; doi:10.1002/eji.201142164)
Supplement: Supplementary file 1 [file eji0042-1767-SD1.pdf]

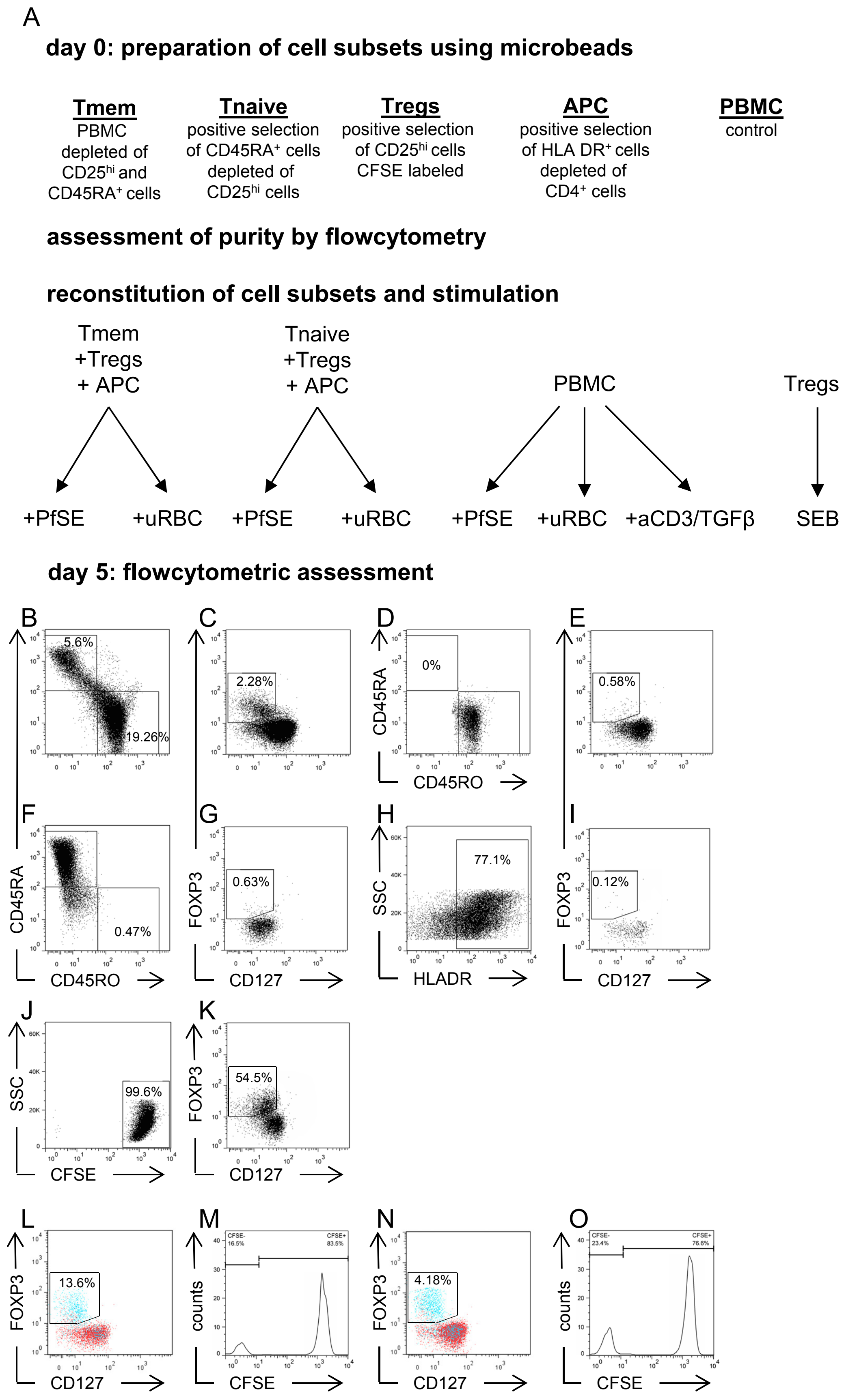
A
day 0: preparation of cell subsets using microbeads
Tmem
PBMC depleted of CD25hi and CD45RA+ cells
Tnaive
positive selection of CD45RA+ cells depleted of CD25hi cells
Tregs
positive selection of CD25hi cells CFSE labeled
APC
positive selection of HLA DR+ cells depleted of CD4+ cells
PBMC
control
assessment of purity by flowcytometry
reconstitution of cell subsets and stimulation
Tmem +Tregs + APC
Tnaive +Tregs + APC
PBMC
Tregs
+PfSE
+uRBC
+PfSE
+uRBC
+PfSE
+uRBC
+aCD3/TGFβ
SEB
day 5: flowcytometric assessment
B
5.6%
19.26%
C
2.28%
D
0%
CD45RA
CD45RO
E
0.58%
F
CD45RA
0.47%
CD45RO
G
FOXP3
0.63%
CD127
H
SSC
77.1%
HLADR
I
FOXP3
0.12%
CD127
J
SSC
99.6%
CFSE
K
FOXP3
54.5%
CD127
L
FOXP3
13.6%
CD127
M
counts
CFSE-
16.5%
CFSE+
83.5%
CFSE
N
FOXP3
4.18%
CD127
O
counts
CFSE-
23.4%
CFSE+
76.6%
CFSE

Suppl. figure 1 Experimental design and cell purities

A scheme of the experimental approach is depicted in Figure (A). Viable $CD4^+$ T cells amongst (B): PBMC, (D): 'Tmem', (F): 'Tnaive' are shown according to their expression of memory markers CD45RA/RO, and according to their expression of Treg markers FOXP3 and CD127 (C: PBMC, E: 'Tmem', G: 'Tnaive', I: 'APC' and K: 'Treg').

Compared to undepleted PBMC 'Tmem' were depleted of > 98% of $CD4^+$ $CD45RA^+$ and > 74% of $CD4^+$ $FOXP3^+$ $CD127^{lo/-}$ T cells. 'Tnaive' cells were depleted > 97% of $CD4^+$ $CD45RO^+$ and > 80% of $CD4^+$ $FOXP3^+$ $CD127^{lo/-}$ T cells compared to PBMC.

(H): the percentage of viable cells expressing HLA-DR amongst 'APC' is shown. The 'APC' fraction comprised of a more than 77% pure $HLADR^+$ population, of which > 94% of $CD4^+$ $FOXP3^+$ $CD127^{lo/-}$ T cells were depleted compared to PBMC. (J): Isolated 'Treg' were uniformly labeled with CFSE and contained at least 54% $CD4^+$ $FOXP3^+$ $CD127^{lo/-}$ cells (K). Note, for better comparability, percentages depicted in figures B-K refer to the proportion amongst viable cells, not $CD4^+$ cells. The proportion of viable $CD4^+$ T cells expressing a Treg phenotype in the cell mix containing (L): 'Tnaive' + 'APC' + 'Treg', or (N): 'Tmem' + 'APC' + 'Treg' is shown; The $CFSE^+$ cells are depicted in blue, $CFSE^-$ cells are shown in red. The histograms in (M) and (O) show the proportion of CFSE+/- cells amongst viable $CD4^+FOXP3^+CD127^{lo/-}$ cells shown in (L) and (N). 'Tnaive' mix (L) and 'Tmem' mix (N) contained a mean of 14.73% and 4.35% CD4 cells expressing a Treg phenotype, respectively, out of which a mean of 17.6% (in the mix with 'Tnaive') and 23.6% (in the mix with 'Tmem') were CFSE neg, indicating the presence of contaminating Treg from either of the non-labelled T cell compartments ('Tmem' or 'Tnaive') or added 'APC'.
